# Mechanical, Electrical, and Thermal Performance of Hemp Fiber-Reinforced Elium Biocomposites Modified with Activated Carbon Nanoparticles: Experiment and Simulation

**DOI:** 10.3390/polym18010066

**Published:** 2025-12-25

**Authors:** Zeenat Akhter, Arvydas Palevicius, Raul Fangueiro, Sultan Ullah, Giedrius Janusas

**Affiliations:** 1 Department of Mechanical Engineering, Faculty of Mechanical Engineering and Design, Kaunas University of Technology, Studentų 56, LT-51424 Kaunas, Lithuania; arvydas.palevicius@ktu.lt (A.P.); sultan.ullah@ktu.edu (S.U.); giedrius.janusas@ktu.lt (G.J.); 2 Fibrenamics, Institute of Innovation on Fibre-Based Materials and Composites, University of Minho, 4800-058 Guimaraes, Portugal; rfangueiro@fibrenamics.com

**Keywords:** hemp fabric, Elium biocomposites, activated carbon nanoparticles, electrical conductivity, tensile properties, FEA, thermal stability

## Abstract

This research examines the influence of various concentrations (0%, 1%, 1.4% and 1.8% by weight) of activated carbon nanoparticles (AC NPs) on the performance of Elium biocomposites reinforced with hemp fibers. Unidirectional [0°/0°] laminates with 20% fiber volume fraction were fabricated via hand layup using two layers of 150 GSM hemp fabric and compression molded to achieve 0.9 mm cured thickness. Quasi-static tensile testing (ASTM D3039, 2 mm/min, 100 mm gauge length) revealed a pronounced non-monotonic relationship between AC NPs loading and mechanical properties, with optimal performance at 1.0 wt.% fillers and catastrophic degradation at 1.8 wt.%. AC NPs filled composites, which were then characterized by their electrical and thermal behavior. Electrically, it also achieved minimum resistivity (1.62 Ω·m) and maximum conductivity (0.62 S·m^−1^), in contrast to the elevated resistance (42.5 kΩ) found in samples with a higher filler content. Thermal analysis showed a slight effect on the degradation of the onset temperature (300 °C) and a higher charring after addition of AC NP. Finite element analysis (FEA) provided a corroboration for these experimental findings, with simulations verification. Microscopy revealed cohesive fractures in the 1.0 wt.% composite whereas voids and brittle failure were evident in samples with higher loading. Hence, the concentration of 1.0 wt.% AC NP offers the best trade off of mechanical, electrical, and thermal properties.

## 1. Introduction

Over the decades, the excellence of composite materials has enabled them to replace historic ones like metals, wood as well as conventional plastics. They are now widely used in aerospace and automobile construction as well as in electronics, farming, and sports equipment [1,2,3,4,5,6]. Hemp fiber is a material, derived out of the Cannabis sativa L. plant, which is becoming popular as a polymer reinforcement accessible within a sustainable economy. By aligning these fibers, unidirectionally (UD) to utilize their natural tensile properties and rigidity, they are created as a viable and clean candidate to replace synthetic fibers such as glass or carbon, since they can be easily produced locally and consume comparatively less power [7,8]. The benefits of UD hemp composites are spread across a family of virtuous properties of being light, economical, significantly acoustic damping, and a source in quick renewable feedstocks. One of the greatest obstacles to their increased use in industry is, however, a basic one, their natural hydrophilicity. This tendency of the fiber to take in moisture causes the polymer matrix bond that holds it to the fiber to become weak, and induces the dimensions. All those are detrimental to the structural integrity and long-term durability of composites based on the composite [9] and constraints of UD hemp composites application to critical load-bearing components to date [10]. Current ongoing research efforts are also concentrated on reducing this drawback through advanced chemical treatment of fibers and modified enhancements on the polymer matrix [11].

Simultaneously, with the emergence of natural fibers, there has arisen the use of activated carbon particles as a disruptive filler material. Due to their miraculous strength-to-mass ratio, high surface area, and remarkable electrical and thermal conductivity, nanoparticles are ideally placed to formulate an advanced composite [12]. Such a distinct property profile has initiated the production of conducting carbon-particle composites in a wide variety of applications to include anti-static surface coatings, aircraft electromagnetic interference enclosure, and advanced sensor technology [13,14,15,16]. Critically, research shows that integrating carbon nanoparticles into natural fiber composites can significantly boost their mechanical performance. For example, Shen et al. [17] found that adding just 0.6 wt.% carbon nanoparticles to a ramie fiber/epoxy composite increased its flexural strength and modulus by approximately 34% and 37%, respectively. Similarly, work by Hongguang Wang et al. [18] reported tensile strength and modulus improvements of 48.7% and 25.2% at 1.0 wt.% MWCNTs. Although there is a difference between activated carbon and CNTs, it provides an idea of homogenizing dispersion with resin for potential electrical properties. Dhilipkumar and Rajesh reported that the addition of 1 wt.% MWCNTs boosted the tensile and flexural strength of co-cured composites to 344 MPa and 379.12 MPa, corresponding to gains of 27.8% and 26.16%. Dehrooyeh et al. [19] confirmed that filler concentration is critical, finding that a 0.5 wt.% CNT loading produced superior tensile strength, Young’s modulus, and toughness over a 1 wt.% formulation. The research community has also directed efforts toward improving both the thermal stability measured via thermogravimetric analysis (TGA/DTG) and the mechanical performance of these hybrid composites [20,21,22]. Recent efforts have, therefore, turned to innovating the matrix itself. The development of sustainable epoxy resins with improved glass transition temperatures targets aerospace demands [23], just as the application of seed oil-derived cationic co-polymers seeks to strengthen adhesion and boost mechanical performance in composites [24]. Hybrid nanofiller use is also another potential option. Research also proves that the synergistic effects of carbon nanotubes and graphene nanoplatelets on an epoxy matrix result in the simultaneous increase in both mechanical strength and electrically conductive properties [25]. Together with this, advancements have been achieved in reinforcement of strong, conductive cellulose-based fibers with MWCNTs which are functionalized to establish many hydrogen bonds [26].

The MAT_124 material model in LS-DYNA is specifically designed for modeling materials that exhibit different behaviors in tension and compression. The model is particularly useful for materials that exhibit asymmetric stress–strain responses under different loading conditions. To date, this model has been successfully applied to modeling polymer composites with synthetic fibers. Appelsved modeled the mechanical behavior of thermoplastic polyamide reinforced with short glass fiber using several models, including MAT_124 [27]. Gurumurthy also employed the MAT_124 model among many adapted numerical models for composites to model a thermoset polymer composite with chopped glass fiber filler [28]. Other researchers used the MAT_124 model for modeling the mechanical properties of polypropylene (PP) with 16% talc as a filler [29]. Activated carbon nano particles (AC NPs) refers to a form of activated carbon with nanoscale particles, known for its high surface area and reactivity. AC NPs properties were taken from the literature and general knowledge of such carbon materials. The skeletal average of density ρ_AC NPs_ = 2000 kg·m^−3^ which excludes pores and represents the solid carbon structure was used [30]. Bulk density (430 kg/m^3^) includes porosity and is unsuitable for rule of mixtures. The Young’s modulus of single AC NPs is difficult to measure directly. For example, one study reported a range of 0.40 GPa to 43.89 GPa for carbon nanoparticles, which represents a wide variation depending on the synthesis method, an assumed value for modeling E_AC-NPs_ = 25 GPa were taken [31].

However, to date, there is no evidence that this model has been applied to modeling polymer composites reinforced with natural fibers. The model is particularly useful for materials that exhibit asymmetric stress–strain responses under different loading conditions [32].

Unidirectional hemp fiber with AC NPs in an Elium thermoplastic works together to produce a high-performance material whose material is cross-sectoral. The biocompatibility of biomedicine, its strength, and lightness can be utilized to design personalized prosthetics and tissue scaffolds in complex shapes to match the conditions of the patient. This high stiffness-to-weight factor that is also useful in medical implants is also useful in the field of engineering, particularly in the area of structural durability because of inherent vibration damping. In addition, the interaction of hemp fibers and nanoparticles yields an enhanced corrosion resistance which presents the prospects of application in the marine and other humid conditions. By including biomedical products as natural-fiber composites integrations, a way to more affordable products are offered, and the existing market views are being challenged. Achieving this goal, however, requires a thorough investigation of the composites’ mechanical, electrical, and functional performance. The composites created using hemp fiber still raise some basic questions, particularly the issue of the polymer matrix in determining the key result or outcome such as strength, conductivity, and heat stability. It is these characteristics that are controllable that will enable application to next-generation sensing applications, including infrastructure, strain gauges, and smart composites with real-time feedback capabilities.

This study investigates a new composite material, which is a uni-directional hemp fiber and activated carbon nanoparticles (AC NPs) in a thermoplastic matrix made of methyl methacrylate (MMA) known as Elium. However, there is no specific published research available on AC NPs-filled Elium resin of unidirectional hemp fiber-reinforced composites for potential electrical, mechanical, and thermal properties. The following studies on related composite systems are presented to provide contextual support and foundational insight for this investigation [33,34,35,36,37,38,39,40,41]. The research provides a comprehensive characterization of its tensile properties supported by the computational modeling of the experimental data, thermal degradation, electrical conductivity, and microstructural features. Future studies will build on this foundation by concentrating on optimizing nanofiller such as CNTs, graphene, etc., dispersion and rigorously assessing the long-term environmental durability of these promising composites.

## 2. Materials and Methods

### 2.1. Materials

In this study, uni-directional hemp fabric at 0° orientation, featuring 150 g/m^2^ was utilized as natural reinforcement materials obtained from the research center of Fibrenamics, Guimaraes, University of Minho, Portugal. While various AC NPs concentration was incorporated into thermoplastic resin known as Elium^®^ 151 XO/SA to fabricate a 2-layer composite. Some of the physical and mechanical properties of natural uni-directional hemp fibers are illustrated in Table 1. Activated carbon nanoparticles (AC NPs) utilized as a nanofiller in this study are characterized by the properties detailed in Table 2. Thermoplastic Elium resin properties are shown in Table 3. All the materials were provided by the research center, Fibrenamics, Guimaraes, Portugal.

The matrix material used for composite fabrication was a thermoplastic Elium resin identified as Elium 151XO/SA and to cure the resin, standard hardener was utilized as the catalyst Butanox M-50. Thermoplastic resin was chosen due to its linear chain and higher mechanical properties, strong fiber adhesion, and durability. While thermoplastic offers moisture resistance and natural fibers absorb moisture due to hydroxyl groups, Elium ensures better interfacial bonding, enhancing load transfer and composite strength.

### 2.2. Fabrication of Composites

The various composites were fabricated via compression molding method, combining a bi-component Elium thermoplastic resin with uni-directional hemp fabric reinforcement. The resin was altered with activated carbon nanoparticle (AC NP) filler at concentrations of 1.0, 1.4, and 1.8 wt.%, with a separate unfilled sample serving as a control. A mechanical stirrer was used to combine the nanoparticles of each formulation in the resin at a speed of 600 rpm for 20 min to obtain an equal dispersion. After this, the hardener (Batanox M-50) was mixed in as per prescribed and manually stirred to begin the reaction of curing.

**Figure 1:**
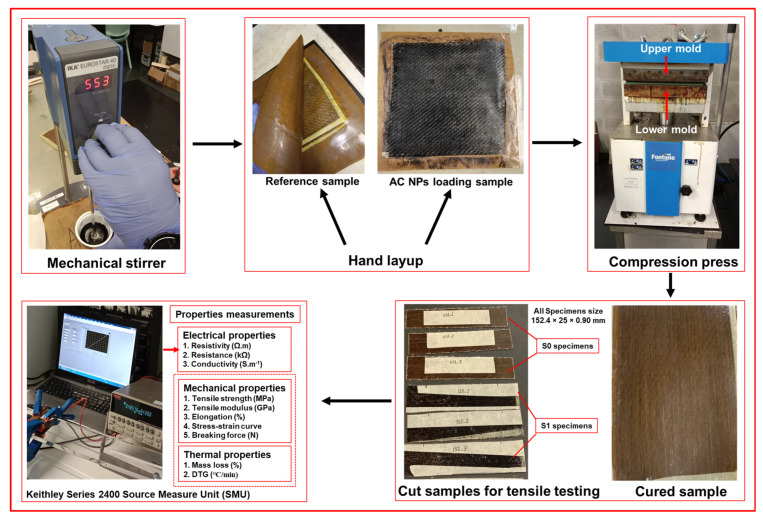
Schematic fabrication procedure.

The catalyzed resin was then applied to two layers of hemp fabric (180 mm × 180 mm) arranged in a [0°/0°] orientation using a hand layup process. The stacked piles were placed in a compression molding machine in between two hot plates and cured under a two-stage pressure cycle: an initial 2 kN pressure was applied for 10 min at 60 °C, followed by an increase to 12 kN for a further 40 min at the same temperature. The cured panels were cooled to room temperature under maintained pressure before demolding. The schematic fabrication procedure is indicated schematically in Figure 1, with detailed formulations and processing parameters summarized in Table 4 and Table 5.

### 2.3. Evaluation and Testing of Composites

#### 2.3.1. Thermogravimetric Analysis (TGA)

The thermal degradation behavior of the composite samples was investigated by thermogravimetric analysis (TGA) at the School of Engineering and the Centre for Textile Science and Technology, Campus de Azurem, University of Minho, Guimaraes, Portugal. All the samples (∼10 mg) were subjected to a temperature ramp from 30 °C to 600 °C at a rate of 10 °C per minute under a 50 mL/min nitrogen purge. This technique assessing mass loss generated three primary datasets: the thermogravimetry (TG) curve tracking mass loss, its derivative (DTG) pinpointing rates of degradation, and a differential scanning calorimetry (DSC) curve revealing associated thermal events.

#### 2.3.2. Electrical Conductivity Test

The bulk electrical conductivity of the composites was assessed through volume resistivity measurements. A Keithley Series 2400 Source Measure Unit (SMU), managed by Kickstart software (2400 Series) at the fibrenamics, Portugal, was used to perform a two-probe method. The current voltage (I V) characteristics were recorded by applying a voltage sweep. The resultant linear plot of I-V which went through the origin showed the behavior of ohmic conduction. The direct values of the inverse slope of the I-V characteristic gave the electrical resistance of the measurement. Volume resistivity (ρ) was subsequently obtained using the expression:

(1) $$\rho =R\times \left(\frac{A}{L}\right)\;$$

where *A* is the cross-sectional area and *L* is the distance between measurement probes. For the specimen geometry used, this relationship simplifies to ρ=R/t, where *t* represents the sample thickness. These resulting resistivity values formed the basis for all subsequent electrical analysis. For statistically significant data and compensation for local material variations, resistance was measured five times on different areas of every sample using a constant 10 mm distance between probes.

#### 2.3.3. Tensile Testing

Tensile tests were carried out on the unfilled reference composite (S0) and the samples containing activated carbon nanoparticles (S1, S1.4, S1.8) using a Hounsfield Tinius Olsen H100KPS Universal Testing Machine at Fibrenamics, Portugal, in compliance with the ASTM D3039 [42] standard. All the specimens, manufactured to dimensions of 152.4 mm by 25 mm with a 0.90 mm thickness, were clamped with a 100 mm gauge length. A crosshead speed of 2 mm/min was applied during testing, and the results documented are the average of five tests for each sample type.

#### 2.3.4. Scanning Electron Microscopy (SEM)

Fracture morphology and damage mechanisms were examined using an FEI Nova 200 NanoSEM (FEG/SEM) [43] at the SEMAT facility, University of Minho, Portugal. To ensure clear imaging in the SEM, the samples were first sputter-coated with 1–3 nm on the sputter-coater model at SEMAT/UM “208HR”.

#### 2.3.5. Modeling of Composites

Numerical modeling was conducted to validate the experimental tensile response of the composites using the Ansys LS-DYNA Suite R16.1 Student software, which is limited to a maximum of 128 × 10^3^ nodes/elements. The specimens were modeled using fully integrated shell elements (ELFORM = 16) with the finite element method. During the tensile test simulation, one end of the specimen was fixed as a rigid boundary. A coarser mesh of 1.31 × 1.25 mm was applied in the grip regions, while a uniform mesh of 1.25 × 1.25 mm was used elsewhere. The tensile specimen consisted of a total of 2400 elements (2541 nodes). Through the thickness of the specimen, seven integration points (NIP = 7) were selected. For the composite specimens, the elasto-viscoplastic MAT_124 tension_compression_plasticity model was chosen, with its parameters provided in Table 6. The MAT_124 material model in LS-DYNA is specifically designed for materials exhibiting distinct behaviors under tension and compression. This model is commonly employed for materials whose yield surface is influenced by hydrostatic pressure (mean stress). The yield surface of this model is the inclined double cylinder that represents the von Mises yield surface, defining the onset of yielding (plastic deformation). It accounts for tension–compression asymmetry, resulting in a direction-dependent plastic response.

The tensile experiments were conducted at a crosshead speed of 2 mm.min^−1^ (corresponding to a strain rate of 3.33 × 10^−4^ s^−1^). Given the prolonged duration of the physical tests, a dynamic numerical simulation was performed using an explicit method, with a selected strain rate of 1 s^−1^. Consequently, the numerical dynamic tensile test was executed at a velocity of 0.1 m. s^−1^, effectively accelerating the process by a factor of 3000. Since the dynamic strain rate remained relatively low, the Cowper–Symonds strain rate sensitivity parameters were omitted in this model. Additionally, viscoplasticity which could have been incorporated via a Prony series, was not modeled. To simulate material failure, multiple approaches were employed. First, within the MAT_124 model, a time-step failure parameter (TDEL) was implemented, primarily to ensure numerical stability. For modeling plastic failure at the required time, an additional MAT_ADD_EROSION model was selected. Given that all tensile specimens exhibited brittle failure, a single erosion criterion (NCS = 1) was applied to the numerical tensile model, using the maximum effective strain at failure (EFFEPS) as the failure criterion. To achieve better alignment between the dynamic model results and the quasi-static test data, the failure parameters in the MAT_ADD_EROSION model were slightly adjusted.

## 3. Results and Discussions

### 3.1. Characterization of Composites

#### 3.1.1. Thermal Analysis of Composites

**Figure 2:**
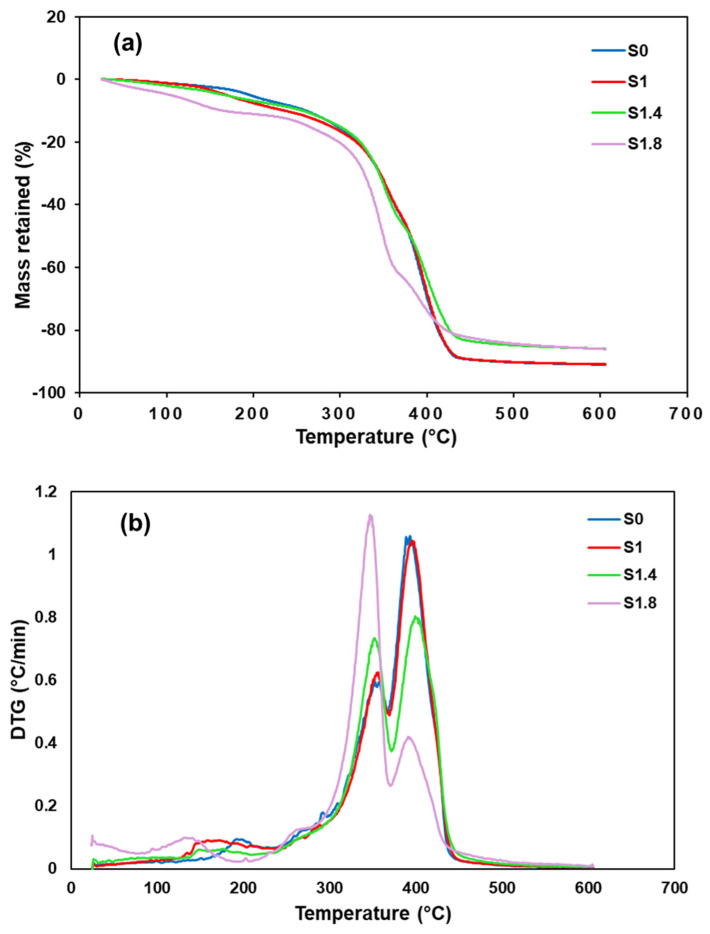
Thermal curves for both reference and AC NPs modified composites (**a**) TG, and (**b**) DTG.

Results from TGA and DTG indicate a substantial improvement in thermal performance for the composites with the addition of activated carbon nanoparticles, as shown in Figure 2. Although all samples start to degrade near 300 °C, those with higher AC NP loadings (1.4% and 1.8%) demonstrate clearly delayed decomposition onset. The DTG thermograms exhibit two principal mass loss steps. The major peak, observed near 400 °C, originates from the concurrent thermal decomposition of the Elium thermoplastic matrix and the hemp fiber reinforcement. When 1.8% AC NPs are added, this peak moves to a higher temperature and becomes less intense, revealing a slower breakdown process and improved thermal stability. This is likely a result of the AC NPs reinforcing the fiber–matrix interface, which restricts the mobility of the polymer chains and retards the degradation process.

The higher residual mass at 600 °C in composites with increased AC NP content points to a protective char layer that limits thermal degradation. This combination of retarded decomposition reduced mass loss rate, and superior char formation substantially increases the material’s suitability for demanding high-temperature environments.

#### 3.1.2. Electrical Properties Measurement of Composites

**Figure 3:**
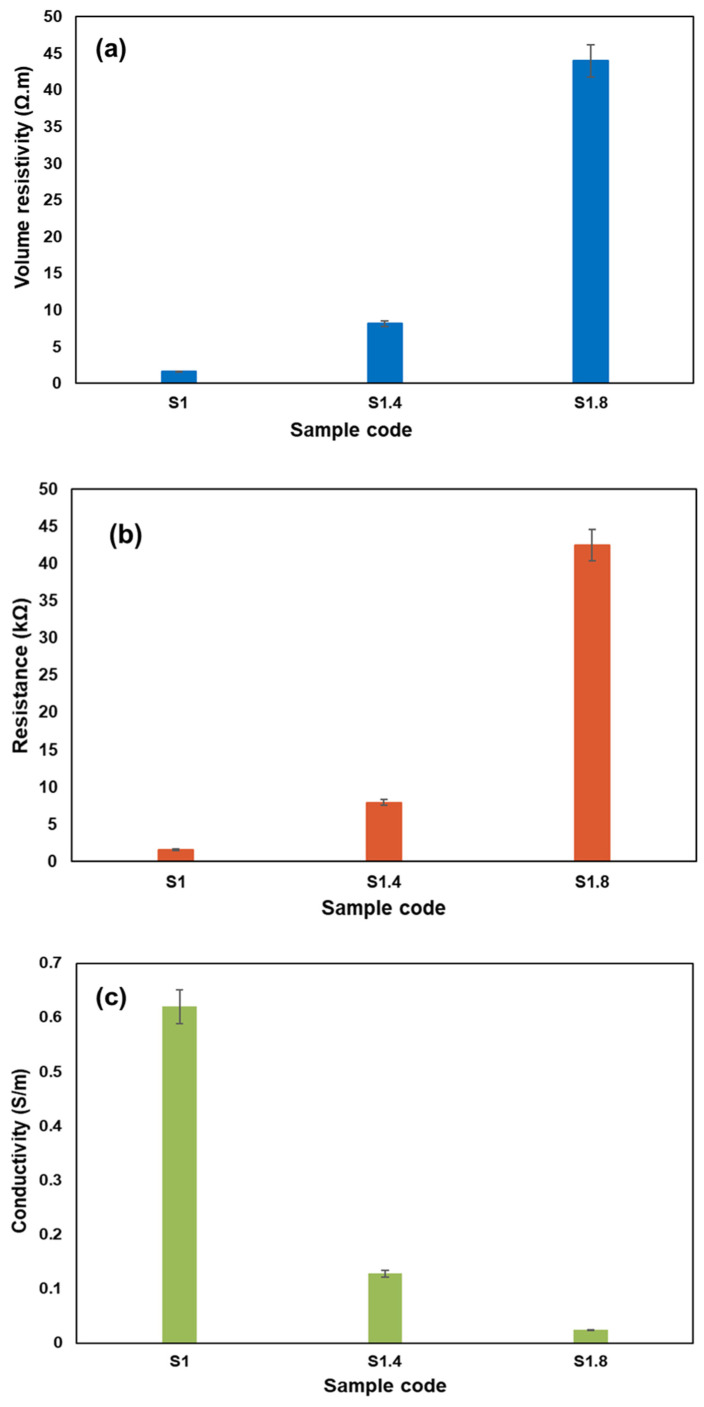
Electrical properties for thermoplastic biocomposites with varying AC NPs content: (**a**) resistivity; (**b**) resistance; and (**c**) conductivity.

The electrical behavior of Elium biocomposites filled with activated carbon nanoparticles (AC NPs) depends on many factors including, type of filler, compatibility of filler with resin, dispersion rate, filler concentration, etc., their resistivity, resistance and conductivity are shown in Figure 3. Adding AC NPs significantly changes the electrically insulating nature of the natural hemp fibers and generating the concept of conductive biocomposites using fabrics as a reinforcement. The 1% AC NP composite (S1) showed the best electrical performance, with a minimum resistivity of ~1.62 Ω·m, a minimum resistance of ~1.57 kΩ, and a maximum conductivity of ~0.62 S/m. These results indicate that this specific concentration allows the nanoparticles to form an interconnected pathway that facilitates electron flow. All electrical measurement data are shown in Table 7.

However, increasing the AC NP content to 1.4% and 1.8% resulted in greater electrical resistivity and resistance, and lower conductivity. The nanoparticles at such high concentrations are prone to agglomeration. These masses of particles separate the continuous conductive networks by playing the role of an insulating region. The nano-particles are arranged due to the physical structure of the uni-directional hemp fibers. Hence, it is important to balance the filler content to come up with natural fiber composites with mechanical strength as well as electrical conductivity.

Mechanical strength and electrical conductivity are improved thanks to the high effectiveness of the combination of the unidirectional hemp fabric with AC NPs. Such a combination of features makes the material ideal in use in systems such as electromagnetic shielding and flexible electronics, where structural integrity as well as consistent electrical performance is required.

The current literature supports this by indicating that incorporation of conductive fillers in natural fiber composites may enhance electrical functioning and at the same time preserve mechanical integrity [44], and as such, they have potential to be the sustainable multifunctional substances [45]. Continued advances in biocomposites based on eco-friendly matrices such as Elium and nanofillers such as activated carbon are laying foundation to a new generation of materials with good structural performance and high electric ability.

#### 3.1.3. Tensile Properties

**Figure 4:**
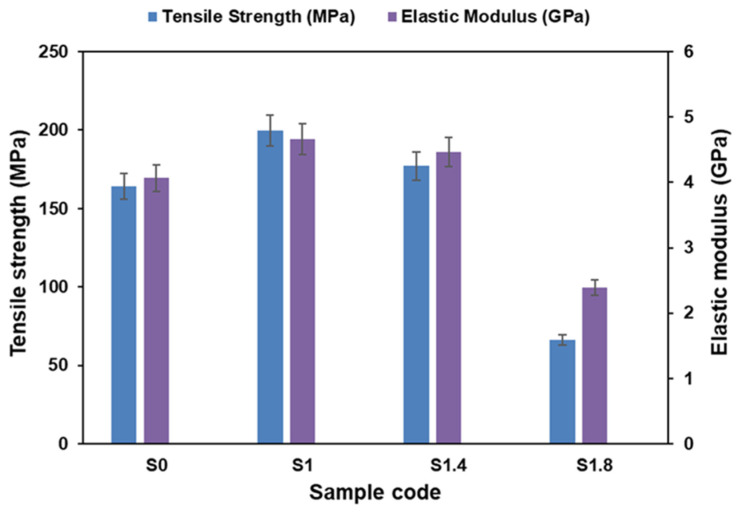
Tensile properties of the hemp/Elium composites with different AC NP concentrations.

Figure 4 shows the tensile behavior of Elium-hemp biocomposites of different content of AC NP. In the case of the unfilled material (S0), the tensile strength was 164.31 MPa and the modulus was 4.06 GPa. These values increased to 199.64 MPa and 4.66 GPa with 1% AC NPs (S1). The improvement in tensile strength and modulus observed for the S1 sample (1% AC NPs) is consistent with previous studies that show low concentrations of nanoparticles enhance interfacial bonding and stress transfer between fibers and the matrix [33,46]. This growth shows that the nanoparticles enhance the adhesion of the fibers to the matrix to achieve a better distribution of mechanical loads.

The 1.4% AC NPs composite (S1.4) presents a reduction in strength to 177.02 MPa and a reduction in modulus to 4.47 GPa as the clumping of the nanoparticles begins to decrease the bond between the fibers and the matrix. At loading of 1.8 (S1.8) performance decreases drastically and strength and modulus reduce to 66.27 MPa and 2.39 GPa. The evidence shows that agglomeration is encouraged by heavy filler. It is these agglomerates that become stress concentrators which weaken the strength of the composite.

These results show that the amount of AC NPs is vital in mechanical performance. Although the optimum reinforcement is 1%, higher reinforcement leads to defects that reduce the properties. In turn, this requires accurate filler dose during sustainable composite design.

**Figure 5:**
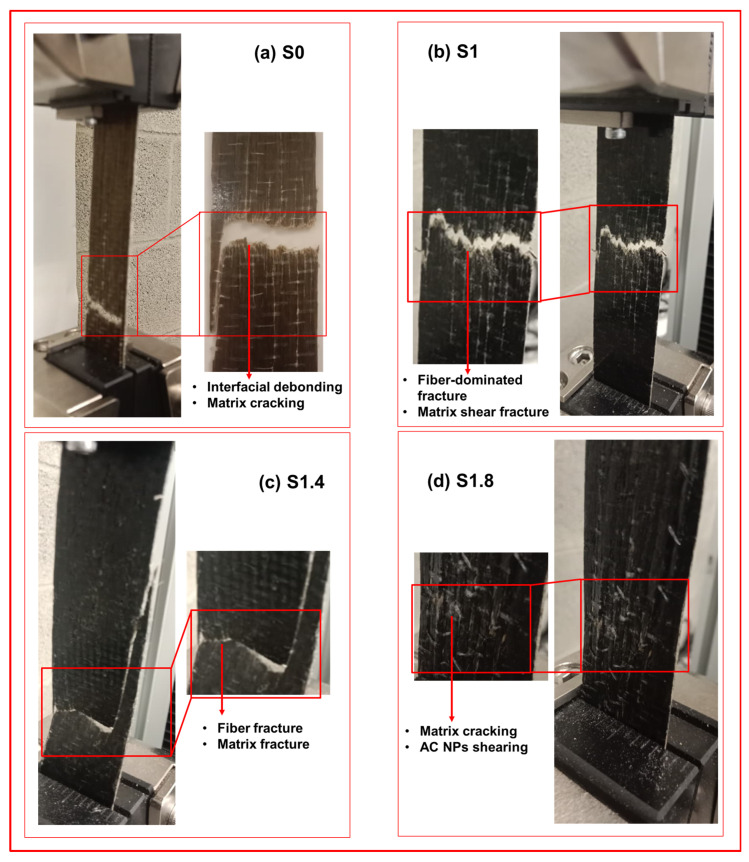
Tensile fractured specimens: (**a**) S0; (**b**) S1; (**c**) S1.4; and (**d**) S1.8.

The surfaces of the fracture in Figure 5 have various failure modes. The straight break of S0 shows low fiber–matrix adhesion whereas the jagged surface of S1 shows high bonding and mixed failure of fibers and resin, following for samples S1.4 and S1.8 with respective details in the Figure. With increased filler content agglomerated nanoparticles cause cracks that propagate at high speeds leading to brittle failure.

#### 3.1.4. Surface and Cross-Sectional Morphological Analysis

**Figure 6:**
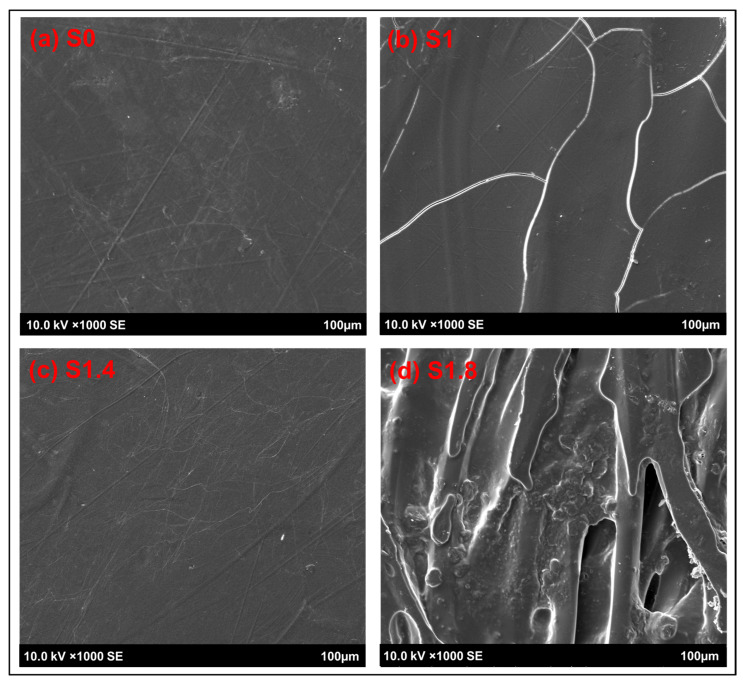
Surface analysis using SEM for the reference sample and AC NPs loaded composites.

**Figure 7:**
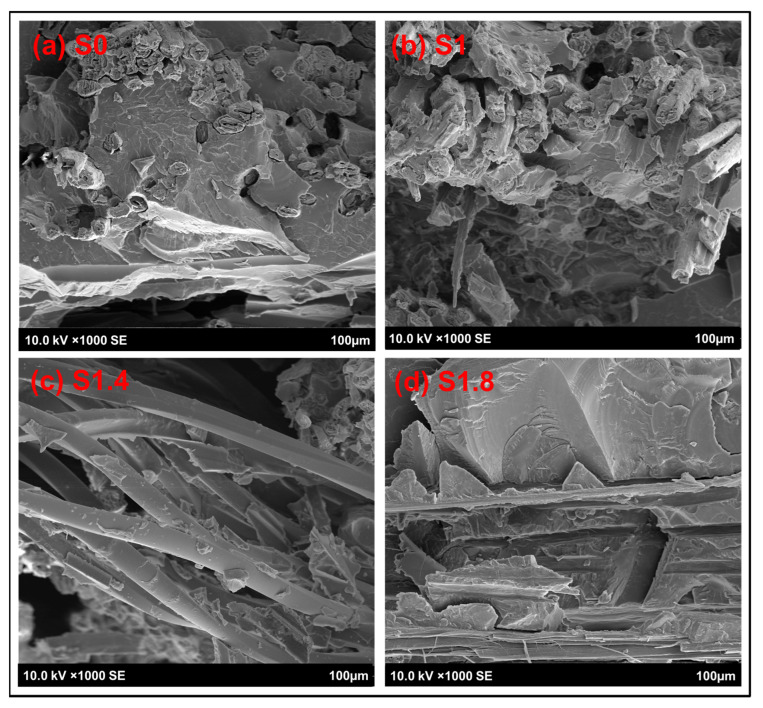
Cross-sectional analysis using SEM for the reference sample and AC NPs loaded composites.

Figure 6 shows the different types of failure: S0 shows smooth fracture with little fiber (weak bonding), S1/S1.4 shows fiber pull-out and microcracks (strong bonding), and S1.8 shows torn resin (brittle failure). These mechanisms of failure are further supported by the cross-sections on Figure 7. The pulled-out fibers and gaps that the S0 specimen has are a sign of low interfacial strength. The transverses of S1 and S1.4 have higher levels of fibers adhering to the matrix and more adhesive fracture than one would expect of materials with high tensile strength. S1.8 reveals fragmented resin and major cracks, confirming that nanoparticle agglomerates create defects that induce brittle fracture.

#### 3.1.5. Experimental Tensile Data Validation by Numerical Simulation and Models

**Figure 8:**
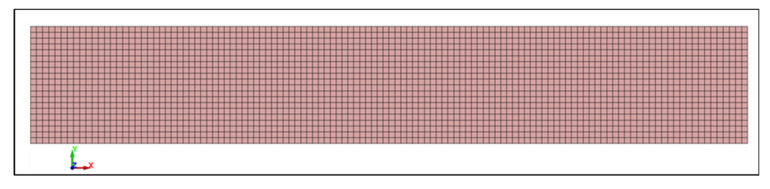
Finite element mesh of the tensile specimen 152.4 mm × 25 mm × 0.9 mm.

**Figure 9:**
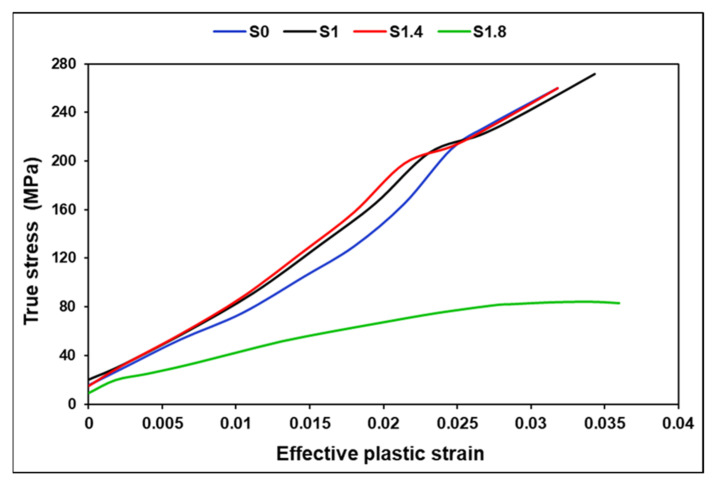
Dynamic tensile LCIDT curves applied during modeling.

A uniaxial tensile test was performed, and its schematic representation is illustrated in Figure 8. The true stress vs. effective plastic strain curves (LCIDT), presented in Figure 9, were derived from the engineering (nominal) stress–strain curves. Table 8 presents the comparison of maximum stress and elongation results obtained during the experiment and numerical tensile modeling.

**Figure 10:**
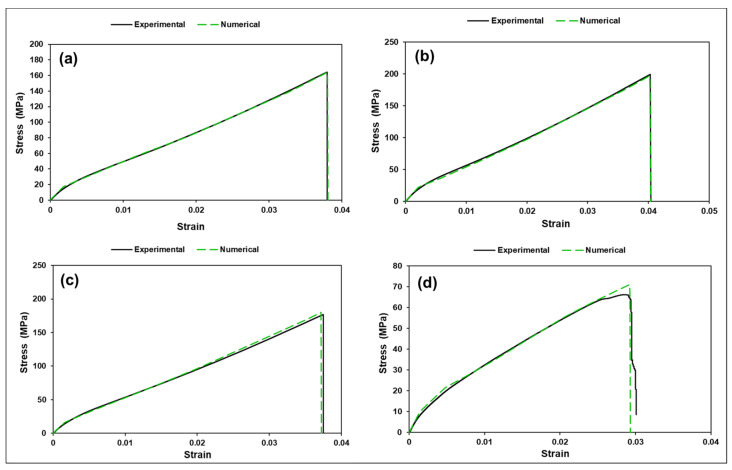
The experimental and numerical tensile test curves (**a**) S0; (**b**) S1; (**c**) S1.4; and (**d**) S1.8.

The experimental and numerical tensile test curves are presented in Figure 10. As can be seen, all composite tensile stress–strain curves coincide with numerical curves with sufficient accuracy. The shape of the deformation curves is similar. Following a short linear region, the curves bend and increase steadily until maximum load is reached. The tensile stress–strain curves are relatively smooth.

With a small powder fraction (S1) at ≈0.58 vol% inside the matrix, the powder can improve local matrix stiffness and (if well dispersed) improve local stress transfer to fibers. Small particulate carbon can act as micro-bridges and reduce microvoid nucleation. That can explain S1 improved ultimate stress (200 MPa) and slightly larger elongation (4.04%) compared to S0 (3.8%) improved load distribution and delayed localization -> higher SED. It is known that small well-dispersed carbon powders can increase strength/modulus if processing yields good wetting [47]. Intermediate powder (S2) at ≈0.81 vol% inside the matrix shows slight performance reduction compared to S1. It may indicate that at 1.4% some degree of particle agglomeration begins. Agglomerates act as stress concentrators and offset any strengthening. Net result: peak strength drops to 177 MPa and elongation is similar to S0 (3.75% compared to 3.80%). High powder (S3) shows strong degradation (drop to 66 MPa). It can be attributed to several reasons such as severe agglomeration forming large local flaws (void-like defects) and impeded wetting of fibers, producing resin-rich or fiber-starved zones or poor interfacial bonding between powder and resin leading to early debonding and microcracking. All of these create large local stress concentrations and early failure though global modulus changed little (our homogenized E values are nearly identical across S0–S3). The large strength drop cannot be explained by small changes in homogenized E. It is a damage/defect/percolation effect: small reinforcing volumes are fine; poor dispersion or processing troubles dominate mechanical strength. With higher concentration of nano powder mechanical reinforcement is unlikely, dispersion and impregnation dominate strength, and powder mainly increases surface area and viscosity, can trap air and produce porosity, and often reduces mechanical strength at higher loadings. This could explain why the S3 result is consistent with porosity and impregnation failure.

**Figure 11:**
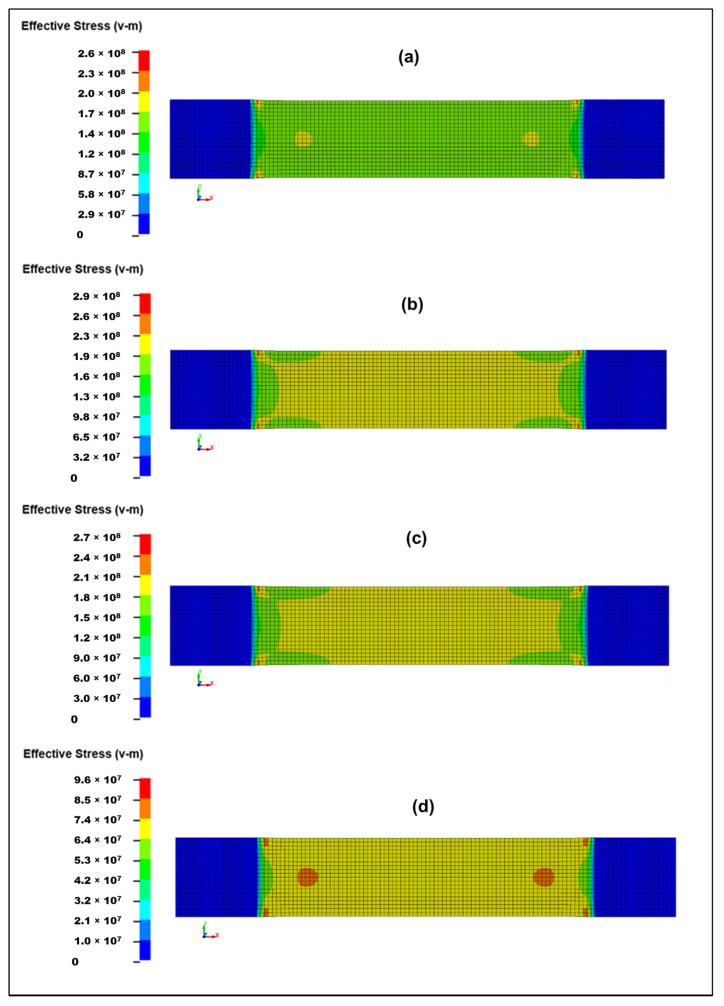
Equivalent von Mises stresses in SI units Pa at 0.01 ms before the start of failure (start of element deletion) after dynamic tensile test simulation: (**a**) S0 (38.02 ms); (**b**) S1 (40.27 ms); (**c**) S1.4 (37.17 ms); and (**d**) S1.8 (29.33 ms).

The von Mises stresses for tensile specimens are illustrated in Figure 11. These trends align well with the experimental tensile behavior and help explain the change from ductile to brittle failure modes across the composite series. All specimens show their maximum von Mises stresses at the specimen shoulders, close to the grip region. This is expected due to the stress concentration induced by constraint and the onset of necking or micro-damage near the transition from clamped to free section. The central gauge length remains mostly uniform in stress, meaning failure is triggered by damage accumulation, not uniform yielding. In addition, in the numerical model, the ends are ideally constrained, whereas the actual constraint differs to some extent. Moreover, the actual specimen exhibits fiber waviness, resin inhomogeneities, surface defects, microcracks, non-uniform matrix–fiber adhesion, regions of varying specimen thickness, and similar imperfections. In the numerical model, the specimen fails at either the fixed or moving end, whereas the actual specimen may fail at the center if a weaker region develops there, despite the fact that stresses at the ends may be somewhat higher. Based on the deformation-strain tensile curves and their agreement with the numerical results, when appropriate composite properties are assigned, the mechanical behavior of such composites can be estimated with sufficient accuracy.

The S0 sample with 0% activated carbon (Figure 11a) has maximum von Mises ≈ 260 MPa. Stress distribution is smooth and extended, meaning the material carries load in a stable, ductile manner. No sharp localization zones appear before failure. S0 shows the most uniform load transfer and stable pre-peak behavior, which corresponds with its relatively high SED and moderate tensile strength. The S1 sample with 1% activated carbon (Figure 11b) has von Mises stresses increase to ~296 MPa, the highest in the series. The distribution is broader than S0, showing load spreading deeper into the gauge section. Shoulders exhibit stronger stress concentration but still without severe localization. S1 demonstrates improved stiffness and load-bearing capacity compared to S0. This matches its highest tensile strength (≈200 MPa) [48] and slightly increased ductility. Activated carbon at this level appears to enhance fiber–matrix load transfer. Distribution remains wide and deep, showing strong resistance to crack initiation. Therefore, 1% activated carbon improves toughness, pre-peak stiffness, energy absorption, fiber–matrix coupling. This aligns with the highest tensile strength (200 MPa). S1.4 sample with 1.4% activated carbon (Figure 11c) has maximum von Mises ~271 MPa, slightly reduced compared to S1. Stress bands appear less wide than S1, slightly more localized towards the shoulders. The transition zone shows early concentration suggesting the beginning of embrittlement. S1.4 shows a partial loss of uniformity, meaning load transfer efficiency reaches an optimum at 1% and decreases when powder concentration becomes excessive. S1.8 sample with 1.8% activated carbon (Figure 11d) has maximum von Mises only ≈187 MPa, the lowest of all. The stress field is highly localized, with small, intense stress hot spots. The gauge region displays weaker stress transmission. S1.8 is significantly more brittle, with poor load-transfer ability. Activated carbon agglomeration likely creates stress raisers, resin-rich or resin-starved zones, and premature crack initiation. This explains the drastic drop in tensile strength (≈66 MPa).

At a concentration of 1% activated carbon (S1), a notable improvement in composite performance is observed. The effective dispersion of the activated carbon particles creates additional interlocking sites, thereby enhancing matrix–fiber bonding. This, in turn, increases the stiffness and tensile peak stress of the composite while simultaneously improving its crack resistance. Conversely, when the activated carbon content exceeds 1.4–1.8% (S1.4–S1.8), a marked deterioration in composite properties becomes apparent. This decline can be attributed to the agglomeration of activated carbon particles, which act as micro-voids, crack initiators, and potential delamination sites within the composite structure. The presence of these agglomerates disrupts the flow of the matrix during the curing process, thereby impairing the effective transfer of stress from the hemp fibers. As a result, the composite exhibits reduced von Mises stress capacity, premature failure, and an overall brittle mechanical response.

## 4. Conclusions

This work analyzes the mechanical, thermal, and electrical behavior of Elium composites reinforced with unidirectional hemp fabric and filled with various concentrations of activated carbon nanoparticles. The properties of Elium-hemp biocomposites change significantly when activated carbon nanoparticles are added. Both experimental tests and computer simulations identified the optimal loading of 1%, AC NPs functions as a multifunctional interface modifier, delivering 21.5% improvement in strength and a 29.2% increase in toughness through synergistic physicochemical mechanisms. The S1 composite (1% ACNP) emerges as the optimal formulation, exhibiting the highest von Mises stress capacity, tensile strength, and ductility. Conversely, the S1.8 composite (1.8% ACNP) represents the poorest performance, characterized by a highly localized von Mises stress field, brittle behavior, and premature failure. While the S0 composite (0% AC NP) performs adequately, it does not match the enhanced stress distribution and energy absorption observed in S1. The strong agreement between numerical results and experimental data (within 0.11–7.28%) further validates the reliability of the model and its applicability for predicting composite behavior. This study highlights a fundamental principle: functional additive design is not merely about increasing additive content but about balancing competing mechanisms at critical thresholds. This insight extends beyond AC NPs to other high-surface-area additives, establishing a framework for the rational design of next-generation sustainable composites that prioritize functional chemistry at interfaces over bulk reinforcement for property enhancement.

The thermal stability of the composites showed gradual enhancement with increased AC NPs content (at 1.8%), demonstrated by a higher decomposition onset temperature (300 °C) and a quantifiable increase of 7% char residue formation. Electrical properties indicated improvement, it also achieved minimum resistivity (1.62 Ω·m) and maximum conductivity (0.62 S·m^−1^), in contrast to the higher resistance observed in samples with higher filler content. The combined results confirm that Elium biocomposites with AC NPs are promising for lightweight, multifunctional structures, but the filler amount is crucial and needs precise control, where both mechanical and electrical conductivity are essential for advanced engineering applications. Future work could test hybrid nanofillers, such as carbon nanotubes mixed with graphene, to seek combined gains in electrical and mechanical performance.

## Figures and Tables

**Table 1:** Physical and mechanical properties of uni-directional hemp fibers.

Properties	Value
Fiber density	1.46 ± 0.02 g/cm^3^
Areal density	150 g/m^2^
Elongation at break	1.5–4%
Water content ^1^	6–12%
Apparent modulus	40–68 GPa

^1^ Valid under the following conditions: 22 °C, 50% RH.

**Table 2:** Properties of activated carbon nano powders (AC NPs).

Properties	Value
Form	Powder
Color	Black
Shape	Cylindrical
Average particle size	<90 nm
Specific surface area	1350 g/m^2^
pH	7.5–10.5
True density	0.41 g/cm^3^
Bulk density	0.32 g/cm^3^
Resistivity	0.2 Ω.cm
Pore size	3–45 nm
Pore volume	1.2–1.4

**Table 3:** Elium 151XO/SA thermoplastic polymer properties.

Properties	Value
Heat deflection temperature	75.90 °C
Tensile strength	47.00 MPa
Tensile modulus	2.6–2.70 GPa
Tensile elongation at break	4.3–4.6%
Flexural strength	80.00–81.50 MPa
Flexural modulus	2.6–2.7 GPa
Flexural elongation	4.42%

**Table 4:** Sample coding and mixing parameters for Elium/hemp composites with varying activated carbon nanoparticle (AC NP) loadings.

Sample Code	AC NPs (%)	Mixer Speed (rpm)	Duration (min)
S0	0	600	20
S1	1	600	20
S1.4	1.4	600	20
S1.8	1.8	600	20

**Table 5:** Details of fabricated samples.

Sample Code	Reinforcement	1st Pressure Cycle (kN)/ Duration (min)	2nd Pressure Cycle (kN)/ Duration (min)
S0	2 layers of hemp (0°/0°)	2/10	12/40
S1	2 layers of hemp (0°/0°)	2/10	12/40
S1.4	2 layers of hemp (0°/0°)	2/10	12/40
S1.8	2 layers of hemp (0°/0°)	2/10	12/40

**Table 6:** Parameters of the MAT_124 model for the numerical tensile testing of composites.

Parameter	Name	Unit	S0	S1	S1.4	S1.8
RO	Mass density	Kg·m^−3^	1120.43	1123.60	1125.55	1128.68
E	Young’s modulus	MPa	8000	9000	8500	6000
PR	Major Poisson’s ratio		0.3545	0.3506	0.3503	0.3503
TDEL	Min time step size for element deletion	s	1 × 10^−10^	1 × 10^−10^	1 × 10^−10^	1 × 10^−10^
EC	Young’s modulus for compression	MPa	8000	9000	8500	6000
RPCT	Scaling factor between E and EC		0.5	0.5	0.5	0.5
PC	Compressive mean stress (pressure)	MPa	5.02	6.76	5.0	3.06
PT	Tensile mean stress (pressure)	MPa	5.02	6.76	5.0	3.06
K	Bulk modulus	MPa	9163.80	10,040.16	9463.37	6680.03

Source: author’s own elaboration.

**Table 7:** Electrical properties of Elium-based biocomposites with varying AC NP concentrations, including resistance, resistivity, and conductivity.

Sample Code	Resistance (Ω)	Std Dev	Resistivity (Ω·m)	Std Dev	Conductivity (S·m^−1^)	Std Dev
S1	1570	116.59	1.62	0.12	0.62	0.05
S1.4	7890	2397.45	8.16	2.48	0.128	0.03
S1.8	42,500	11,792.18	44.0	12.2	0.0239	0.01

**Table 8:** Comparison of maximum stress σ_max_ and elongation ε_max_ results obtained by experiment and numerical tensile modeling.

Sample	Maximum Stress σ_max_ (MPa)			Maximum Strain ε_max_ (%)		
	Experiment	Numerical	Diff. %	Experiment	Numerical	Diff. %
S0	164.31	163.99	−0.20	3.80	3.81	0.24
S1	199.64	197.12	−1.27	4.040	4.036	−0.11
S1.4	177.02	179.77	1.55	3.75	3.72	0.74
S1.8	66.27	71.09	7.28	3.01	2.93	2.59

## Data Availability

The original contributions presented in this study are included in the article. Further inquiries can be directed to the corresponding author.
